# Trends in Appendicitis and Perforated Appendicitis Prevalence in Children in the United States, 2001-2015

**DOI:** 10.1001/jamanetworkopen.2020.23484

**Published:** 2020-10-30

**Authors:** Darryl T. Gray, Trina Mizrahi

**Affiliations:** 1 Center for Quality Improvement and Patient Safety, Agency for Healthcare Research and Quality, Rockville, Maryland

## Abstract

This sequential, cross-sectional study examines the prevalence of perforated appendicitis in children in the United States in relation to access to health care from 2001 to 2015.

## Introduction

Appendiceal perforation is potentially avoidable with timely recognition and appropriate treatment of acute appendicitis. Therefore, perforated appendicitis may indicate suboptimal access to quality health care.^1,2,3^

## Methods

This sequential, cross-sectional study independently captured longitudinal samples of aggregated deidentified administrative data. Therefore, the Agency for Healthcare Research and Quality’s Human Subjects Protection Officer institutionally designated it as exempt from the need for formal institutional review board review or patient consent. This study followed the Strengthening the Reporting of Observational Studies in Epidemiology (STROBE) reporting guideline. The State Inpatient Databases (SIDs) of the Agency for Healthcare Research and Quality’s Healthcare Cost and Utilization Project (HCUP) capture administrative data on discharges from nonfederal hospitals in participating states.^4^ HCUP’s internally accessible disparities analytic files (DAFs) capture SID discharges meeting specific criteria for race/ethnicity coding.^5^ However, US hospitals do not report patient vs institutional identification of race/ethnicity on submission of SID data. We identified DAF discharges among children aged 1 to 17 years (January 1, 2001, to September 30, 2015) who had appendicitis and, more specifically, appendiceal perforation specifically (codes are in the eAppendix in the Supplement^3^). By applying these codes in the HCUP’s online query system (HCUPnet: https://hcupnet.ahrq.gov), we also estimated nationwide volumes of perforated and nonperforated pediatric appendicitis discharges (January 1, 2000, to December 31, 2014) based on HCUP’s annual National Inpatient Sample (NIS) and its triennial Kids’ Inpatient Database (KID). These databases sample approximately 20% and approximately 80% of annual discharges recorded in SIDs, respectively. Two-sided *P* < .05 was considered statistically significant. Analyses were performed using Excel 2016 (Microsoft, Inc) and SAS, version 9.2 (SAS Institute Inc).

## Results

**Figure 1.**
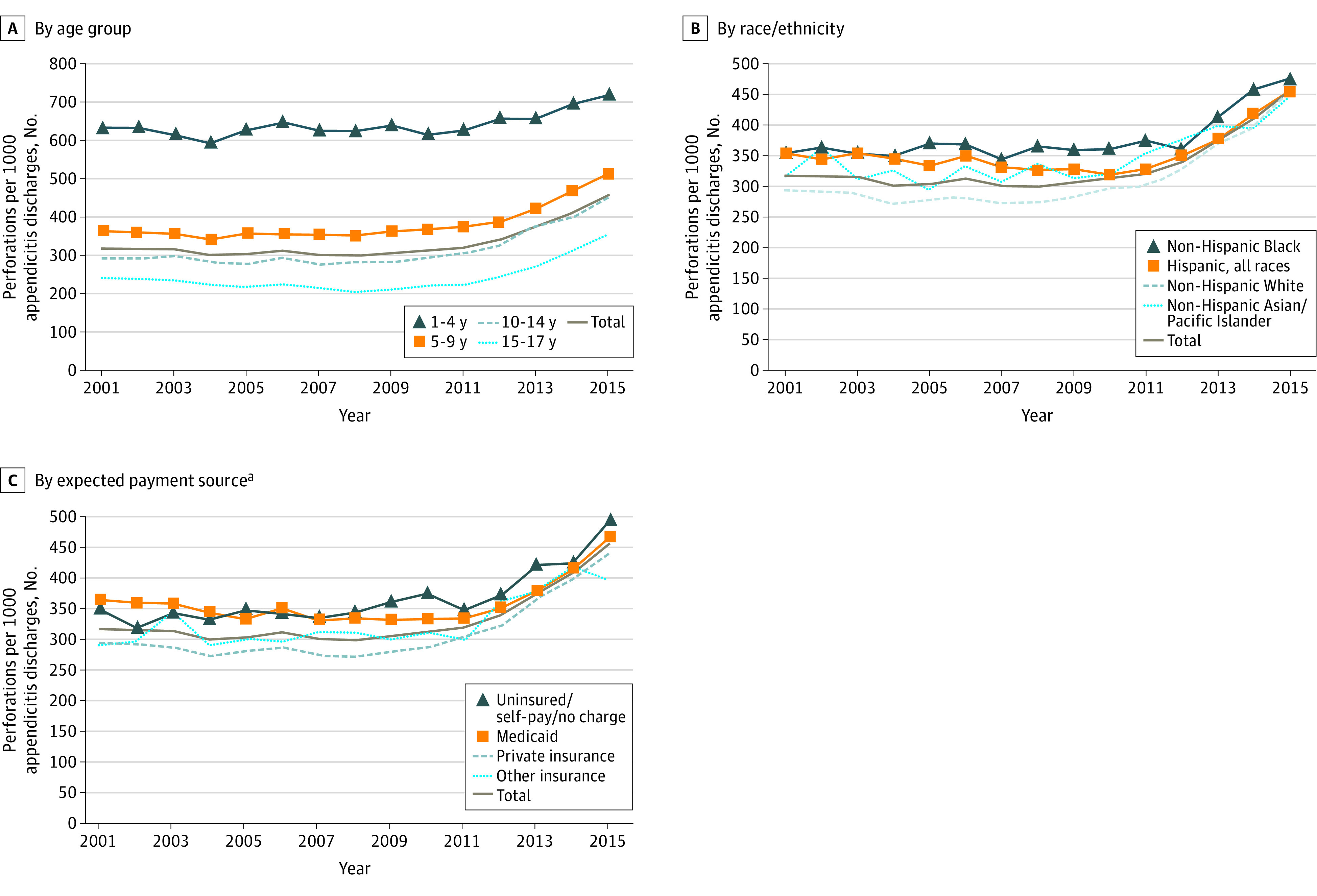
Perforated Appendicitis Cases in US Children Aged 1 to 17 Years With Appendicitis Perforated appendicitis cases per 1000 discharges of children aged 1 to 17 years with appendicitis by age group (A), race/ethnicity (B), and expected payment source (C) (disparities analytic file data). ^a^Data from the small and atypical pediatric Medicare population were excluded.

**Figure 2.**
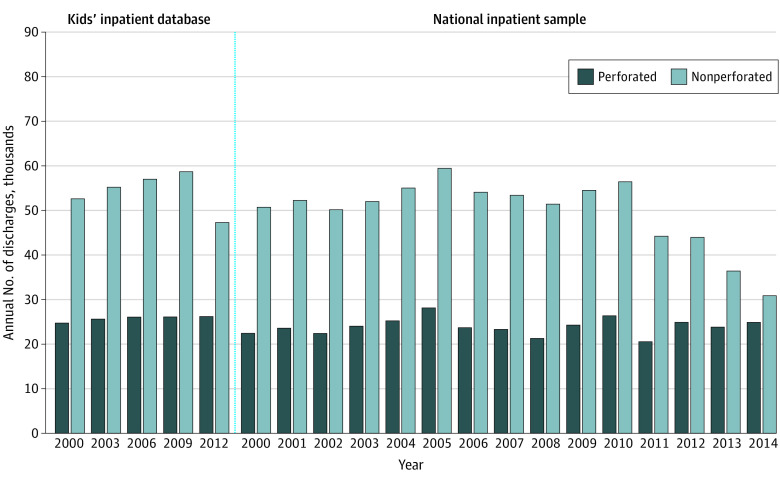
Annual Volumes of Perforated and Nonperforated Appendicitis Cases in US Children Aged 1 to 17 Years Data were obtained from the Kids’ Inpatient Database and National Inpatient Sample.

Annual sample sizes of appendicitis case counts in DAFs ranged from 15 414 (January 1 to September 30, 2015) to 35 415 (January 1 to December 31, 2009). Perforation rates rose from 317.5 per 1000 appendicitis cases in 2001 to 457.7 per 1000 cases in 2015, with consistent gradients by age groups (Figure 1A). Rates for boys and girls were similar (eg, 320.2 vs 316.1 per 1000 cases in 2001; 2-sided *P* = .65 [*z*-test] and 452.1 vs 458.6 per 1000 cases in 2015; *P* = .79). Differences across some sociodemographic groups narrowed. For example, rates for Black and White patients were reviewed as a potential measure of variable care access. This gap narrowed from 357.0 per 1000 cases vs 294.4 per 1000 cases (*P* < .005) in 2001 to 476.7 per 1000 cases vs 457.7 per 1000 cases (*P* = .33) in 2015 (Figure 1B). However, uninsured/self-pay/no charge patients had consistently higher rates than did privately insured patients (eg, 350.9 per 1000 cases vs 295.8 per 1000 cases in 2001 [*P* < .005] and 496.5 per 1000 cases vs 443.0 per 1000 cases in 2015 [*P* = .04]) (Figure 2A). Hospitals in large metropolitan core areas originally had higher perforation rates than did hospitals in “noncore” (ie, rural) areas (eg, 354.1 per 1000 cases vs 269.9 per 1000 cases in 2001; *P* < .005) and 317.8 per 1000 cases vs 244.0 per 1000 cases in 2009; *P* < .005). By 2015, rates in both settings had risen to essentially the same level (465.8 per 1000 cases vs 462.2 per 1000 cases; *P* = .91).

The NIS annual sample sizes of appendicitis case counts ranged from 11 068 (January 1 to December 31, 2014) to 18 395 (January 1 to December 31, 2005). The KID’s counts ranged from 40 584 (January 1 to December 31, 2000) to 58 071 (January 1 to December 31, 2009). Upweighted NIS and KID estimated nationwide volumes of pediatric perforations were approximately 25 000 cases per year (Figure 2). Decreasing estimated nonperforated appendicitis case volumes seen after 2009-2010 produced net increases in observed perforation rates when perforations were expressed as proportions of all appendicitis cases.

## Discussion

The results of this sequential, cross-sectional study indicate that perforation rates based on DAF data rose over time. Age-based differences seen in a given year presumably reflect age-related variations in sign/symptom reporting.^6^ However, concurrent variation seen across sociodemographic subgroups could reflect differential access to appropriate care.^2^ The NIS and KID appendicitis data showed stable perforation case volumes, accompanied by unanticipated decreasing volumes of nonperforated appendicitis cases. Thus, NIS and KID perforation rates rose, mirroring DAF results. We hypothesize that perforation rate increases over time may reflect increasingly accurate exclusion of nonappendicitis cases from perforation rate denominators, rather than declines in nonperforated appendicitis frequencies.

Our nationwide data identified intriguing trends. However, study limitations included data lags, potential coding inaccuracies, and limited clinical information. In addition, various desired multivariate analyses could not be performed on aggregate DAF data. Also, HCUPnet data in which multiple diagnosis codes are combined do not include CIs or statistical testing options. Limitations of DAF and HCUPnet data precluded performing our entire analysis on a single data set.

Future analyses of encounter-level administrative and/or clinical data could include consideration of the potential effects on perforation rates of factors such as coding variation (including changes related to use of the *International Statistical Classification of Diseases and Related Health Problems, Tenth Revision*), shifts in appendicitis epidemiology or in imaging and treatment patterns, and outcomes such as appendicitis-negative appendectomies. Such analyses could address our post hoc hypothesis, otherwise extend our findings, and potentially prompt reconsideration of the current perforation rate indicator.

## Notes

Supplement. **eAppendix.** Established *ICD-9-CM* Principal or Secondary Discharge Diagnosis Codes **eReference.**

## References

[1] Jablonski KA, Guagliardo MF Pediatric appendicitis rupture rate: a national indicator of disparities in healthcare access. Popul Health Metr. 2005;3(1):4. doi:10.1186/1478-7954-3-4 15871740 PMC1156944

[2] Baxter KJ, Nguyen HTMH, Wulkan ML, Raval MV Association of health care utilization with rates of perforated appendicitis in children 18 years or younger. JAMA Surg. 2018;153(6):544-550. doi:10.1001/jamasurg.2017.5316 29387882 PMC5875324

[3] Agency for Healthcare Research and Quality Pediatric quality indicator 17 (PDI 17): perforated appendix admission rate. Published August 2018 Accessed August 2, 2020. https://www.qualityindicators.ahrq.gov/Downloads/Modules/PDI/V60/TechSpecs/PDI_17_Perforated_Appendix_Admission_Rate.pdf

[4] Agency for Healthcare Research and Quality Healthcare Cost and Utilization (HCUP) Project. Last updated July 2020 Accessed August 2, 2020. https://www.hcup-us.ahrq.gov/

[5] Barrett M, Coffey R, Houchens R, Heslin K, Moles E, Coenen N Methods applying AHRQ quality indicators to Healthcare Cost and Utilization Project (HCUP) data for the 2017 National Healthcare Quality and Disparities Report (QDR). HCUP Methods Series Report No. 2018-01. Published May 2018 Accessed August 2, 2020. https://www.hcup-us.ahrq.gov/reports/methods/2018-01.pdf

[6] Paulson EK, Kalady MF, Pappas TN Clinical practice: suspected appendicitis. N Engl J Med. 2003;348(3):236-242. doi:10.1056/NEJMcp013351 12529465

