# Supplementary material for: Trends in Appendicitis and Perforated Appendicitis Prevalence in Children in the United States, 2001-2015
Source: JAMA Netw Open. 2020 Oct 30;3(10):e2023484. doi: 10.1001/jamanetworkopen.2020.23484 (PMC7599446; doi:10.1001/jamanetworkopen.2020.23484)
Supplement: Supplement. — eAppendix. Established ICD-9-CM Principal or Secondary Discharge Diagnosis Codes eReference. [file jamanetwopen-e2023484-s001.pdf]

Supplemental Online Content

Gray DT, Mizrahi T. Trends in appendicitis and perforated appendicitis prevalence in children in the United States, 2001-2015. *JAMA Netw Open*. 2020;3(10):e2023484.
doi:10.1001/jamanetworkopen.2020.23484

eAppendix. Established *ICD-9-CM* Principal or Secondary Discharge Diagnosis Codes

eReference.

This supplemental material has been provided by the authors to give readers additional information about their work.

© 2020 Gray DT et al. *JAMA Network Open.*

eAppendix. Established[1] *ICD-9-CM* principal or secondary discharge diagnosis codes

Appendicitis
540.0 acute appendicitis with peritonitis
540.1 abscess of appendix
540.9 acute appendicitis not otherwise specified
541 appendicitis not otherwise specified

Appendiceal Perforation
540.0 acute appendicitis with peritonitis
540.1 abscess of appendix

eReference

1. Agency for Healthcare Research and Quality. Pediatric quality indicator 17 (PDI 17): perforated appendix admission rate. Published August 2018. Accessed August 2, 2020. https://www.qualityindicators.ahrq.gov/Downloads/Modules/PDI/V60/TechSpecs/PDI_17_Perforated_Appendix_Admission_Rate.pdf

© 2020 Gray DT et al. *JAMA Network Open.*
